# Freezing and freeing of degrees of freedom in joint action learning

**DOI:** 10.3389/fpsyg.2023.1287148

**Published:** 2023-11-23

**Authors:** Marijn S. J. Hafkamp, Remy Casanova, Reinoud J. Bootsma

**Affiliations:** Institut des Sciences du Mouvement, Aix-Marseille Université, CNRS, Marseille, France

**Keywords:** joint action, interpersonal coordination, collaboration, motor learning, motor control, degrees of freedom

## Abstract

In daily life, we often encounter situations in which we have to coordinate our actions with others to achieve a common goal. These actions are also defined as joint actions. In this study we investigated how a multi-agent system learns to acquire control in a novel joint action task. To this end, we designed a task in which agents had to coordinate their actions so as to control a ball rolling on a long, hand-held beam. Participants' task was to roll the ball as fast and accurately as possible back-and-forth between two indicated targets on the beam, by manually adjusting the inclination angle of the beam. In the joint action version of this task, two participants each hold a different beam extremity. In a solo action version, the participant holds one extremity while the other is attached to a static support. The experiment consisted of two practice sessions that each comprised 15 two-min trials. One group of 12 participants first performed a solo action session of the task and then a joint action session (Group S/J), while another group of 12 participants started with a joint action session, followed by a solo action session (Group J/S). While performance increased over practice in all sessions, we found that in the joint action task dyads without prior solo task experience (Group J/S) adopted a sequential pattern of interpersonal coordination by freezing their motion whenever the other agent moved. In contrast, dyads that had received prior practice in the solo task setting (Group S/J) demonstrated less freezing and more complementary motion during the joint action performance. Lastly, we found that initial practice as a dyad in the joint action task did not result in a significant improvement of a subsequent solo action performance. We concluded that multi-agent motor learning in a novel joint action task is characterized by the initial freezing of task-relevant degrees of freedom, while individual training in a constrained setting can stimulate the freeing of these DFs during subsequent joint action performance.

## 1 Introduction

Humans are social animals. In many of our daily activities we coordinate our actions not only with the physical events or objects that surround us, but also with the actions and intentions of others. Whether we perform a well-practiced dance choreography with a partner or spontaneously help a friend to carry a heavy box, interpersonal coordination is omnipresent and often essential for task success. Tasks in which the coordination between agents is required to achieve a common goal are also known as joint actions (Sebanz et al., 2006; Butterfill, 2012). During a joint action, collaborating individuals are interdependent. For example, when two agents carry a box together, the functionality of each individual's actions –such as lifting, lowering or rotating the box– is dependent on the actions of the partner. This dependency couples the individual movement systems and creates a functional multi-agent system (Marsh et al., 2006, 2009; Riley et al., 2011). Within this system, agents not only share the goal of carrying a box, but they also share the control over the box. Generally, the acquiring of control in a task involves a process of motor learning, in which an individual progresses over practice from task novice toward task expert (Bernstein, 1967). The current study aimed at characterizing motor learning in a joint action task, in which two agents are in the process of acquiring collective control in their task environment. To do this, we designed a challenging joint action task in which agents had to learn to coordinate their actions so as to control a ball rolling on a hand-held beam.

Interpersonal coordination can be approached as a systemic phenomenon that emerges from the interactions between agents and their environment (Marsh et al., 2009; Riley et al., 2011; Schmidt et al., 2011). For instance, when people are rocking chairs side-by-side, they are spontaneously attracted to a state of synchronous rocking (Richardson et al., 2007). This state of interpersonal coordination is not planned or orchestrated, but it emerges from the visual coupling between the agents and it is constrained by the properties of the system, such as the natural periods of the chairs (cf. Richardson et al., 2007). Such spontaneous coordination of action between agents can in fact emerge from different modes of coupling, whether they are mechanical (e.g., Harrison and Richardson, 2009) or informational (see Néda et al., 2000; for an example of auditory coupling and Van der Wel et al., 2011; for an example of haptic coupling). This phenomenon shows how lawfulness in interpersonal coordination is to be found at the level of the emergent behavior of the multi-agent system, rather than at the level of the processing of information of the individual agents (Marsh et al., 2009). In the following, we refer to this perspective as the systems approach to interpersonal coordination.

In the example of chair rocking (Richardson et al., 2007) interpersonal coordination emerges unintentionally and without a purpose. We defined a joint action task, however, as a task in which interpersonal coordination is required to achieve a common goal. To accommodate for this functional dimension of interpersonal coordination, Riley et al. (2011) proposed the Interpersonal Synergies Theory (IST). IST provides a systems account of coordination between agents who are involved in a joint action. The central concept in this framework is that of a synergy. A synergy is a functional grouping of degrees of freedom (DFs) of a system that temporarily acts as a coordinated unit (Bernstein, 1967; Turvey, 2007; Kelso, 2009, 2022). The core claim of IST is that during joint action these synergies not only emerge *within* individual movement systems, but also *between* two or more movement systems, underlying successful interpersonal coordination. For example, interpersonal synergies between the body segments of two individuals are assumed to underly coordination in the act of carrying a box together, as exemplified earlier.

Arguably, to date the most direct empirical support for the IST account of joint action comes from the bimanual pointer-and-target aiming paradigm, in which two agents together have to bring a pointer and a target to coincide (Mottet et al., 2001; Romero et al., 2015). Mottet et al. (2001) demonstrated that the end-point variance of the relative motion between the pointer and the target was smaller than the sum of the end-point variances of these two effectors. This difference increased when accuracy demands of the task were higher, suggesting a functional interpersonal coupling –or synergy– between the pointer and the target operated by the two individuals. Using the same task paradigm, Romero et al. (2015) showed that not only the end-effectors, but also the joint angles of both individuals compensated for each other to stabilize the relative pointer-to-target motion. Moreover, in a static version of this interpersonal aiming task Ramenzoni et al. (2012) showed that considering the body segments of both individuals as the DFs of one multi-agent system resulted in a larger dimensional compression than considering the body segments as the DFs of two independent movement systems. This implies a stronger functional coupling of DFs between individuals than within individuals, adding to the evidence for the emergence of interpersonal synergies during joint action.

Manual pointing, however, is a relatively simple task for which practice is not required to be sufficiently coordinated. To illustrate, in the study of Romero et al. (2015) successful pointing of collaborating dyads was achieved in all trials and practice merely led to a small decrease in movement time. In contrast, Rigoli et al. (2015) investigated a joint action task in which task control was considerably more difficult to achieve. In their experiment, two agents jointly controlled a virtual ball rolling on a virtual labyrinth platform, by each manipulating a different joystick to tilt the platform. Rigoli et al. (2015) analyzed the performances on the first two trials and found that collaborating dyads spontaneously and mutually adapted to different joystick-to-platform mappings. By limiting their analysis to the first trials, they highlighted the spontaneous and adaptive character of the interpersonal coordination, confirming the findings of Mottet et al. (2001) and Romero et al. (2015) in the pointer-and-target paradigm. Yet, due to the mediating role of gravity and other passive forces in the system, jointly controlling a rolling ball on a tilted labyrinth is much more challenging than bringing a pointer and a target together.

Research in the domain of individual motor learning has demonstrated that patterns of coordination can change significantly and qualitatively over practice when a task is sufficiently challenging (e.g., Vereijken et al., 1997). This raises the question whether multi-agent systems also display qualitative changes in interpersonal coordination over learning. Findings of qualitative shifts in interpersonal coordination in a multi-agent shepherding task (Nalepka et al., 2017) suggest that this might indeed be the case. Therefore, the goal of this study was to characterize a multi-agent motor learning process. To this end, we designed the continuous manually-controlled ball-and-beam task, a new joint action version of the (small-scale) manual ball on beam positioning task explored by Huang et al. (2006).

**Figure 1:**
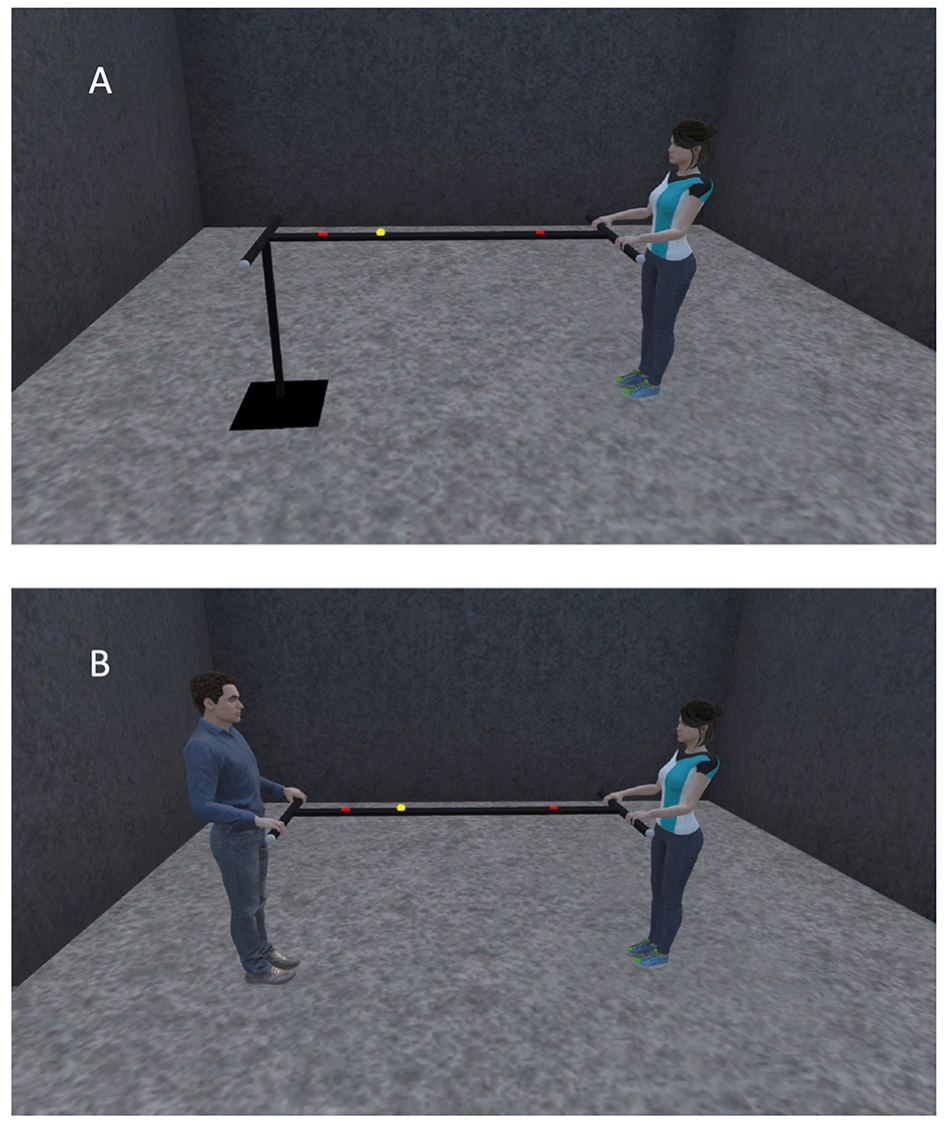
Schematic illustration of the ball-and-beam task configuration used in the solo action task setting **(A)** and in the joint action task setting **(B)**.

Our manual ball-and-beam task is a novel and challenging perceptuomotor task in which motion of a ball on a long hand-held beam is controlled by adjusting the inclination of the beam. The goal of the task is to roll the ball as fast and accurately as possible back-and forth between two targets on the beam. Thus, our task design contains elements of both Fitts' classical reciprocal aiming task (Fitts, 1954; Mottet and Bootsma, 1999: Mottet et al., 2001) and gravity-mediated tasks such as virtual labyrinth ball rolling (Rigoli et al., 2015), inverted-pendulum aiming (Van der Wel et al., 2011) and pole balancing (Foo et al., 2000; Jacobs et al., 2012). The difficulty of the task lies in the non-linear relationship between the motions of the beam and the ball, with the latter governed by the gravitational and other passive forces in the system. The manual ball-and-beam task can be performed alone (i.e., solo action task setting, Figure 1A) as well as together (i.e., joint action task setting, Figure 1B). In the solo action task setting, one end of the beam is statically supported while the other extremity can be manipulated by the agent. In the joint action task setting, on the other hand, the inclination of the beam can be adjusted independently by each of the two agents. In other words, in the joint action version, the ball-and-beam system as a whole has two systemic, that is, task-level specific, DFs. The question we address in this contribution is how these two DFs –and hence the two collaborating agents– are coordinated when they learn to control the ball together. In this contribution we therefore focus on the task related changes observed over practice in the solo and joint action settings.

According to Bernstein's (1967) theory of individual motor learning, the first stage of motor skill learning is characterized by the freezing of redundant DFs in the movement system, which he defined as the biomechanical joints of the body. By decreasing the motion around one or more joints, a novice freezes DFs to acquire initial control in a challenging task (Vereijken et al., 1992). Over learning, these DFs are progressively released, which ultimately allows performance-beneficial exploitation of the passive forces in the system without losing control. Due to the exploitation of such passive forces, the expenditure of active forces by the agent decreases, creating a more efficient task execution (e.g., Sevrez et al., 2012). In general, this theory of motor learning is well supported in the literature (see Guimarães et al., 2020). Freezing of joint angles during the first stage of learning has been found in for instance dart throwing (McDonald et al., 1989; Didier et al., 2013), ski-simulation movements (Vereijken et al., 1992; Hong and Newell, 2006) and football kicking (Anderson and Sidaway, 1994; Hodges et al., 2005). Newell and Vaillancourt (2001), on the other hand, proposed to interpret Bernstein's recruitment of DFs as a more abstract adaptation in the dimensionality of the attractor dynamic in the movement system rather than as an increase in recruitment of biomechanical joint angles *per se*. Underlying this proposal was the idea that DFs of a system are only relevant for learning when they are considered in relation to the demands of the specific task at hand (see also Gray, 2020). Our choice to focus on the coordination of the two systemic DFs of the ball-and-beam system, that is the two agents' contributions to beam motion, rather than on the coordination of the joint angles of each individual, is in line with this idea.

In this general context, we expected that the first stage of a multi-agent motor learning process would be characterized by the freezing of systemic DFs in the ball-and-beam system. More concretely, our first hypothesis was that the motion of one or both of the two individuals in a dyad would initially be minimized to create a more controllable system. Secondly, we hypothesized that individual agents' prior task experience in a constrained task setting would stimulate a multi-agent system to free-up its DFs by releasing the constraints. To test this second hypothesis, we designed an experiment in which participants not only performed the ball-and-beam task as a dyad in the joint action task setting, but also as an individual in the solo action task setting where there is only one systemic DF available. We expected that dyads with prior individual experience in such a constrained (1 DF) solo action setting would more readily display freeing-up of DFs in the (2 DF) joint action setting than dyads without any such individual task experience. We therefore asked one group of participants to first participate individually in a solo action task session before participating in a joint action task session (Group S/J). A second group of participants performed the joint action task session without prior practice on the solo action task. In order to evaluate whether joint action task experience was, in turn, beneficial to solo action task performance, the second group of participants (Group J/S) subsequently participated in a solo action task session.

## 2 Materials and methods

### 2.1 Participants

A group of 24 students and junior staff members from Aix Marseille University (9 women, 15 men, with an average age of *M* ± *SD*: 24.1 ± 3.7 years) participated voluntarily in this study. All participants were free from known motor impairments and reported normal or corrected-to-normal vision. Before the start of the first experimental session the participants were informed about the aim and procedure of the experiment. All participants provided written informed consent before participating in the study. The study was approved by the French National Ethics Committee for Research in Sports and Movement Sciences (CERSTAPS) and conducted according to University regulations and the Declaration of Helsinki.

### 2.2 Task and procedure

The task equipment consisted of a standard golf ball (4.3 cm diameter, 46 g weight) that could roll over a 2-m long, 3 by 3 cm V-shaped iron beam, with tubular (length 54 cm, diameter 3 cm) handles perpendicularly attached to its extremities. Participants' task was to roll the ball as fast and accurately as possible back-and-forth between two 15-cm wide targets clearly visible on the beam. Participants were instructed that they would receive one point for every time that the ball reversed direction within a target's boundaries and that their goal was to achieve as many points as possible within a 2-min trial. With both target centers located 60 cm from the beam's midpoint, the center-to-center inter-target distance was 120 cm. Bimanually holding one handle allowed a participant to lift or lower the beam's extremity and thereby change its inclination angle. In the solo action task setting, one of the two handles was attached to a pivot on a static pole, whose height was adjusted to the participant's hip height. In the joint action task setting each handle was hand-held by a different participant. In order to compensate for more than 10 cm height differences between participants, the smaller participant stood on a wide and stable wooden platform that allowed 2-cm stepwise height adjustments.

The experiment consisted of two sessions of practice on the ball-and-beam task completed on two different days, with 1 or 2 days between the sessions. The 24 participants were randomly divided into two groups of 12 participants. One group first performed a solo action session and then a joint action session (group S/J). The other group first performed a joint action session and then a solo action session (group J/S). Participants within each group were randomly paired into dyads for the joint action sessions. Each session began with a short warm-up trial of ~1 min, during which participants were given the chance to familiarize themselves with the ball-and-beam task. This was followed by 15 two-min practice trials, divided in blocks of three trials. Blocks were separated by 2–3 min breaks. During the trials, participants were not allowed to talk or communicate otherwise. Talking was allowed during the breaks but could not concern the task.

At the start of each trial, the ball was placed in the middle of the beam. Verbal signals from the experimenter signaled the beginning (“Go”) and the end (“Stop”) of a trial. After each trial, participants received verbal feedback on their score, based on experimenter observation. Participants were regularly encouraged to try to improve their score on each new trial, in order to reach the highest score possible within a session. In both the solo and the joint action task practice session participants competed indirectly with the other players via a leaderboard that expressed the high scores of the other participants. Moreover, small (delicacy) prizes were awarded to those with the highest scores. All of this was intended to motivate participants to perform optimally on each new trial. Participants were asked to refrain from communicating with others about task strategies or any other aspects of the ball-and-beam task.

### 2.3 Data acquisition and analysis

All sessions took place in a darkened room. To capture the motion of the ball and the beam, a reflective marker was attached to each handle and the ball was chosen to be bright yellow. Participants wore dark clothes and black gloves to ensure sufficient contrast with the ball and the markers. All trials were filmed with a tripod-mounted GoPro9 camera that was positioned at a 1-m height and a 2-m perpendicular distance from the midpoint of the beam. This allowed ensuring that all relevant ball and beam motion occurred well within the field of view. The camera was set in linear lens mode so as to obtain minimal image deformation. In the joint action task setting, participants were asked to take place on fixed locations 2.5 m apart, holding the beam in between them. All trials were recorded with a (recalculated) sample frequency of 59.94 Hz and a resolution of 2.7K. Due to technical issues, six trials were excluded from analyses, leaving a total of 534 trials for analysis. No session had more than one missing trial.

Video recordings of all trials were imported into Kinovea (https://www.kinovea.org/), which was used to track the two-dimensional (X, Y) motion of the ball and the two reflective markers on the beam extremities. X and Y position time series were then analyzed using MATLAB. All data were first filtered using a fourth-order low-pass Butterworth filter (cut-off frequency of 3 Hz) and trimmed to the 120-sec trial duration. The ball trajectory was subsequently transformed from motion in a two-dimensional space to one-dimensional motion along the beam, represented by the motion along the virtual line between the two markers, with the inter-marker distance used for calibration. The ball motion and the motion of the two markers on the beam extremities formed the basis of all further analyses. We analyzed the data on three distinct levels of the multi-agent system: (1) the level of task performance, (2) the level of beam inclination and (3) the level of interpersonal coordination.

The level of task performance comprised an analysis of the ball motion. Using a peak finding algorithm, we split the ball position timeseries into separate cycles and counted the number of ball reversals within the target zones to calculate the scores per trial. Average ball speed over a trial was calculated by multiplying the ball cycle frequency with twice the average amplitude of the ball cycles. Thirdly, ball accuracy was expressed in terms of the effective target width reached in a trial. This variable was calculated by multiplying the standard deviation of the ball position peaks around the two targets by 1.96 (Welford, 1968). Lower effective target width therefore indicated higher ball accuracy on a trial.

The second level of the analysis concerned the beam inclination angle. This variable represents the “end-effector” of the ball-beam system, that is, the variable that is manipulated so as to control the ball rolling on the beam. Note that in the joint action version of the task, beam inclination is influenced by the motion of both individuals and thus a result of their underlying coordination. The beam inclination angle was calculated at each time step by taking the inverse tangent (atan) of the X-Y positions of the two beam extremity markers. From these beam inclination angle time-series we derived an average beam inclination amplitude per trial by multiplying the average absolute inclination angle with π/2.

Lastly, the third level of the analysis comprised an analysis of the interpersonal coordination. For this, we used the vertical motion of the two beam extremities. We calculated the proportions of the trial times that both participants moved in the same direction (referred to as the supplementarity ratio), in the opposite direction (referred to as the complementarity ratio) or that at least one of the two individuals moved slower than a threshold value of 0.05 m/s (referred to as the freezing ratio). To investigate whether or not the observed freezing was equally divided over the two individuals in a dyad, we calculated individual freezing ratios as well, defined as the proportion of the time that vertical beam velocity of each individual was lower than 0.05 m/s.

### 2.4 Statistical analysis

For the purpose of statistical analysis, we set the data for the missing trials (6 out of 540) to the average value of the 14 remaining trials of that participant (4) or dyad (2) in the corresponding session.

While the participants performing the joint action session as dyads also performed the solo action individually, for the present purposes we did not seek to relate dyads' performance on the joint action task to the corresponding individuals' performance on the solo action task. This led us to adopt a design for the statistical analyses with two groups of twelve participants for the solo action task and two groups of six dyads for the joint action task. A 2 x 2 x 15 mixed-design ANOVA, with the between-participant factors Task (solo action, joint action) and Group (S/J, J/S) and the within-participant factor Practice (15 trials), was used to analyze Task, Group, and Practice effects on dependent variables performance score, average ball speed, effective target width and average beam inclination amplitude, calculated for each trial. For the joint action task setting, interpersonal coordination indicators complementarity ratio, supplementarity ratio and freezing ratio were analyzed using a 2 x 15 mixed-design ANOVA, with the between-participant factor Group (J/S, S/J) and the within-participant factor Practice (15 trials).

For all analyses the significance level was set to α = 0.05. Greenhouse-Geisser corrections were applied when violations of the sphericity assumption were detected. *Post-hoc* tests were performed using Tukey HSD. Where appropriate means and standard deviations are reported as *M* ± *SD*.

## 3 Results

### 3.1 Task performance

#### 3.1.1 Performance score

**Figure 2:**
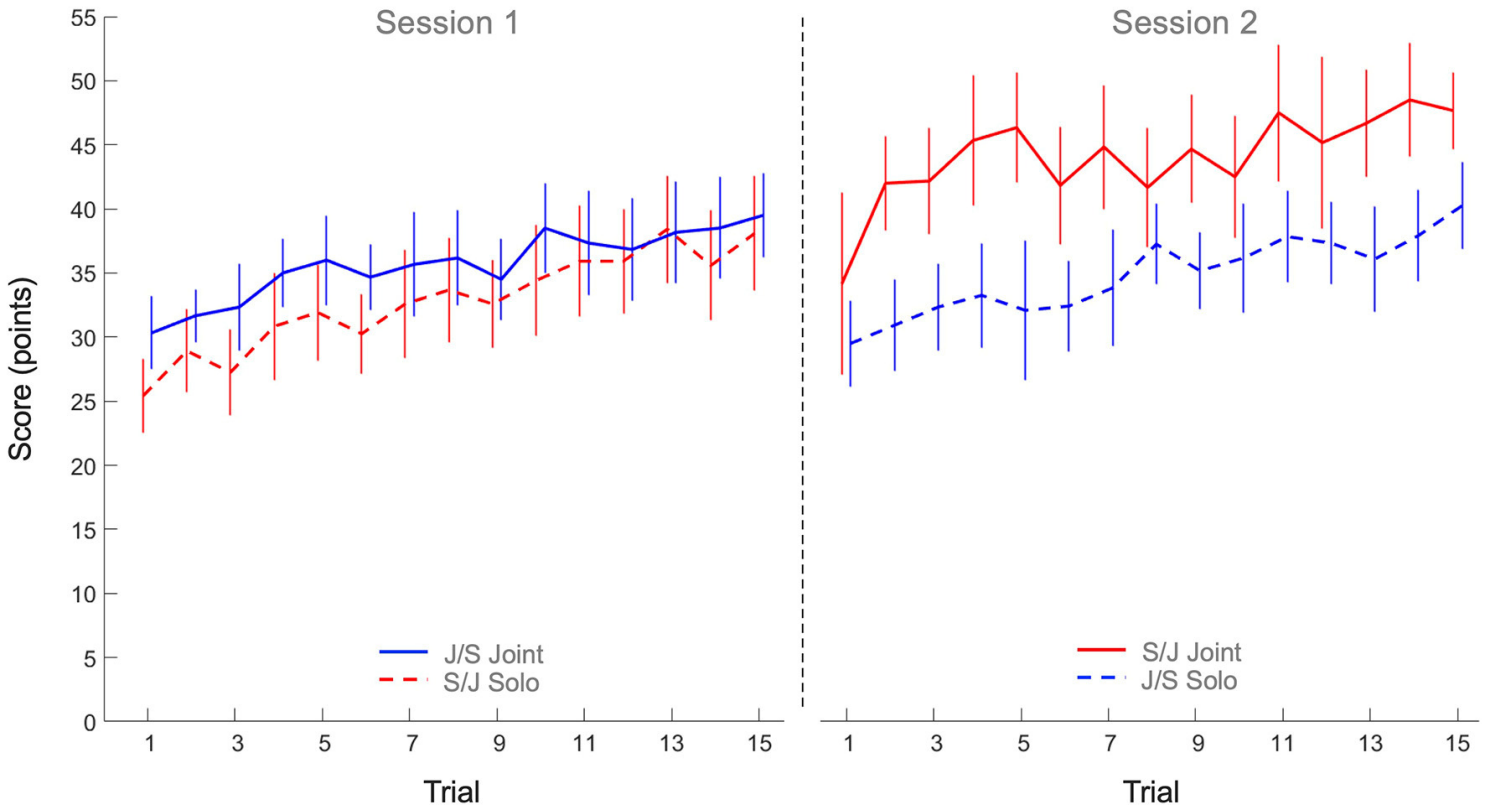
Mean performance scores (points) over practice trials per group and per task. Solid lines indicate joint action task performance, while dashed lines represent solo action task performances. Group S/J (red) first performed the solo action task (Session 1) and then the joint action task (Session 2), whereas Group J/S (blue) first performed the joint action task (Session 1) and then the solo action task (Session 2). Error bars represent between-participant standard deviations.

The 2 x 2 x 15 mixed design ANOVA on performance score revealed significant main effects of Practice (*F*(7.53, 240.98) = 16.62, *p* < 0.001, η^2^_*p*_ = 0.34) and Task (*F*(1, 32) = 6.44, *p* = 0.016, η^2^_*p*_ = 0.17), as well as a significant Task x Group interaction (*F*(1, 32) = 4.75, *p* = 0.037, η^2^_*p*_ = 0.13). All other effects were not significant. As can be seen from Figure 2, the main effect of Practice indicated that both groups improved their scores over trials on both tasks. *Post hoc* analysis of the Task x Group interaction revealed that the joint action score of Group S/J (44.1 ± 7.6 points) was significantly higher than the solo scores of Group J/S (34.8 ± 6.6 points, *p* = 0.047) and Group S/J (32.8 ± 7.0 points, *p* = 0.011), but not significantly higher than the joint action score of Group J/S (35.7 ± 5.8 points, *p* = 0.160). No other differences were significant.

#### 3.1.2 Ball speed

**Figure 3:**
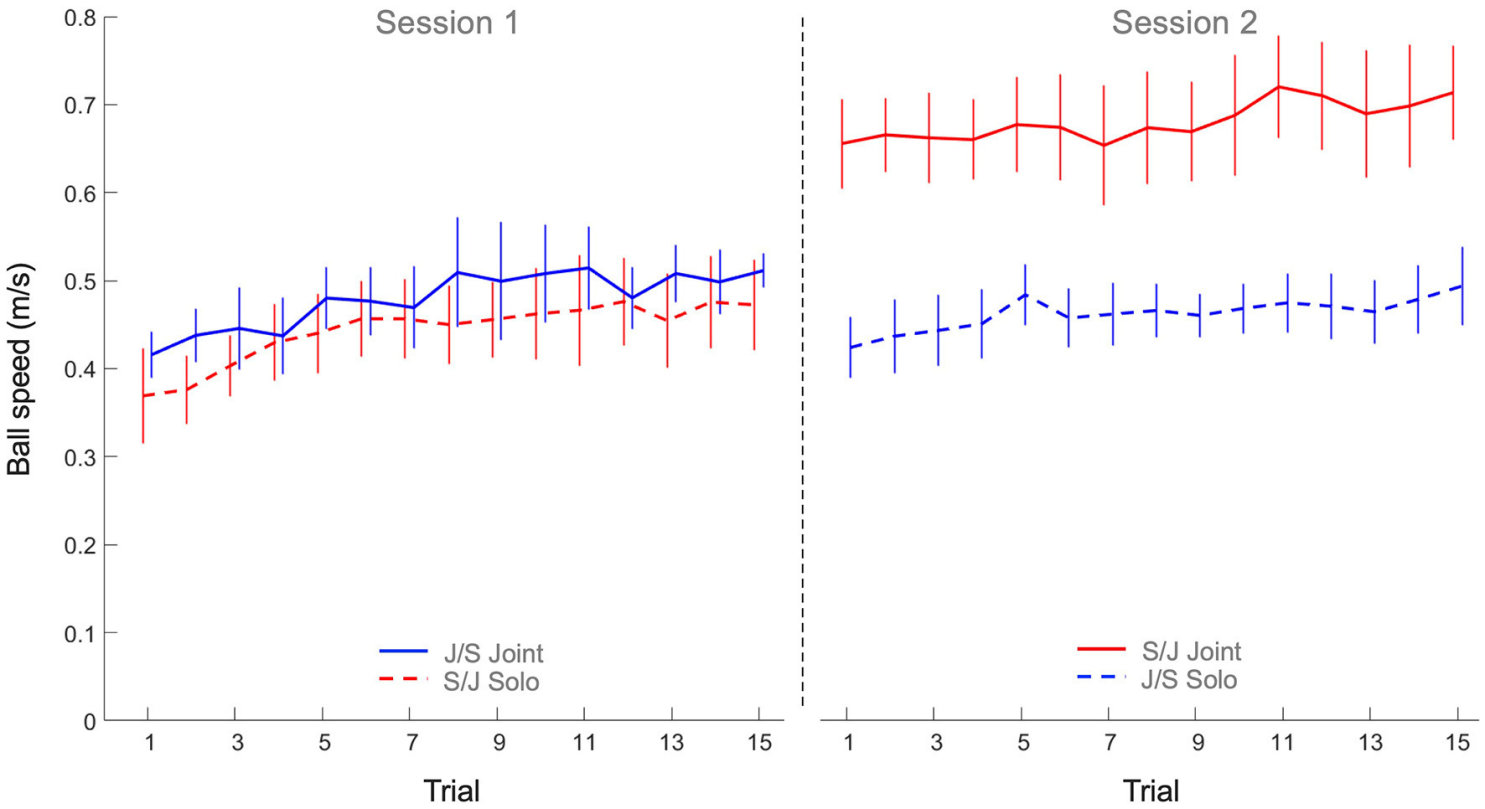
Mean ball speed (m/s) practice trials per group and per task. Solid lines indicate joint action task performance, while dashed lines represent solo action task performances. Group S/J (red) first performed the solo action task (Session 1) and then the joint action task (Session 2), whereas Group J/S (blue) first performed the joint action task (Session 1) and then the solo action task (Session 2). Error bars represent between-participant standard deviations.

The 2 x 2 x 15 mixed design ANOVA on average ball speed revealed significant main effects of Practice (*F*(4.42, 141.53) = 8.61, *p* < 0.001, η^2^_*p*_ = 0.21), Task (*F*(1, 32) = 20.6, *p* < 0.001, η^2^_*p*_ = 0.39) and Group (*F*(1, 32) = 10.5, *p* = 0.003, η^2^_*p*_ = 0.25), as well as a significant Task x Group interaction (*F*(1, 32) = 15.5, *p* < 0.001, η^2^_*p*_ = 0.33). All other effects were not significant. As can be seen from Figure 3, the main effect of Practice indicated that both groups increased their ball speed over trials on both tasks. *Post hoc* analysis of the Task x Group interaction revealed that Group S/J displayed a significantly (*p* < 0.001) higher ball speed on the joint action task (0.68 ± 0.11 m/s) than Group J/S (0.48 ± 0.08 m/s). Group S/J's ball speed on the joint action task was moreover significantly higher than the ball speed on the solo action task of Group S/J (0.44 ± 0.08 m/s, *p* < 0.001) and Group J/S (0.46 ± 0.06 m/s, *p* < 0.001). No other differences were significant.

#### 3.1.3 Ball accuracy: effective target width

**Figure 4:**
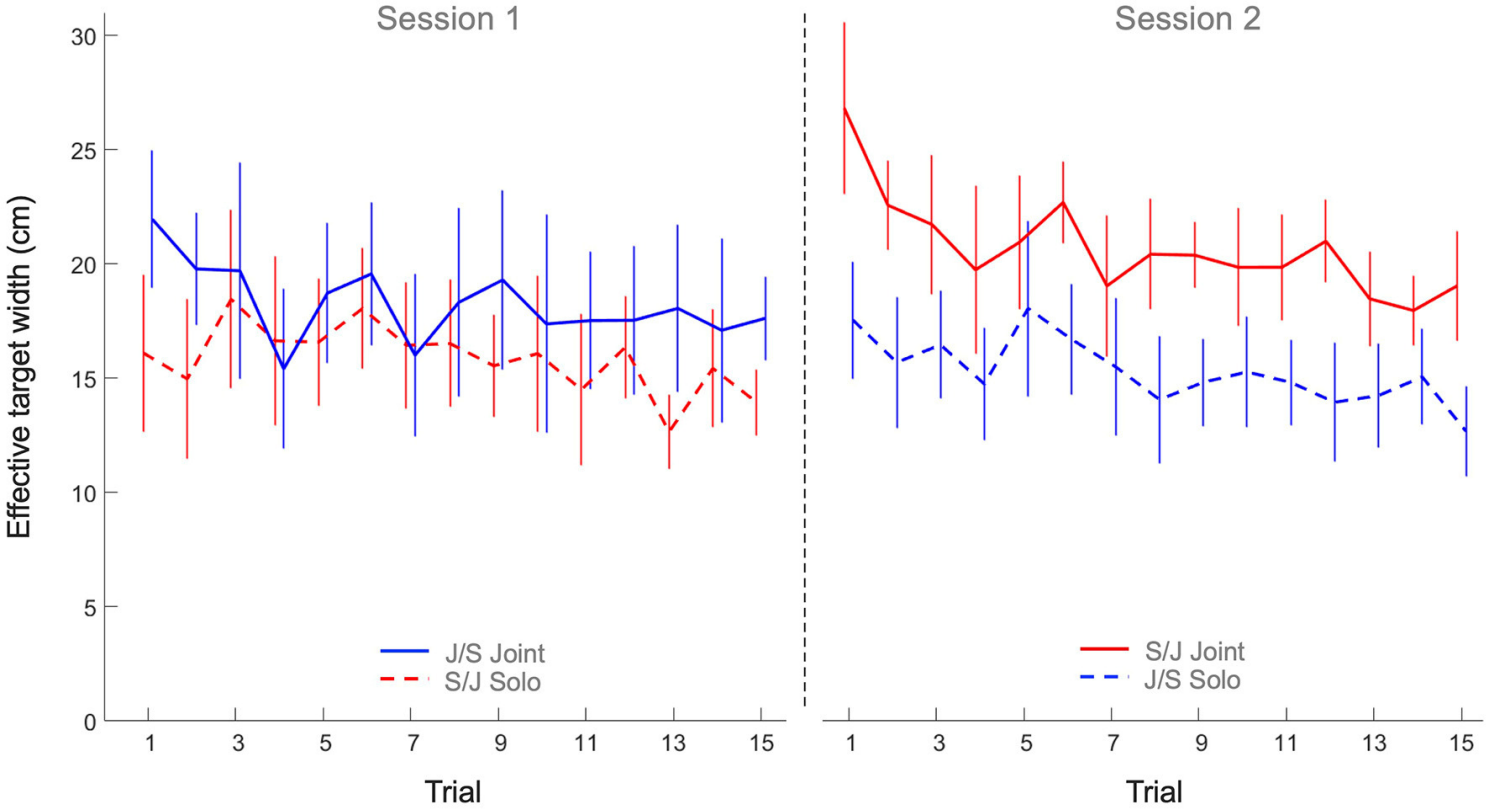
Mean effective target width (cm) over practice trials per group and per task. Solid lines indicate joint action task performance, while dashed lines represent solo action task performances. Group S/J (red) first performed the solo action task (Session 1) and then the joint action task (Session 2), whereas Group J/S (blue) first performed the joint action task (Session 1) and then the solo action task (Session 2). Error bars represent between-participant standard deviations.

The 2 x 2 x 15 mixed design ANOVA on effective target width revealed significant main effects of Practice (*F*(7.10, 227.16) = 3.36, *p* < 0.001, η^2^_*p*_ = 0.10) and Task (*F*(1, 32) = 7.57, *p* < 0.010, η^2^_*p*_ = 0.19). All other effects were not significant. As can be seen from Figure 4, the main effect of Practice indicated that both groups decreased their effective target width (i.e., increased their ball accuracy) over trials on both tasks. The main effect of Task, on the other hand, indicated that the effective target width of participants in both groups was lower on the solo action task (15.6 ± 3.4 cm) than on the joint action task (19.5 ± 4.9 cm), indicating a general higher ball accuracy on the solo action task.

### 3.2 Beam inclination amplitude

**Figure 5:**
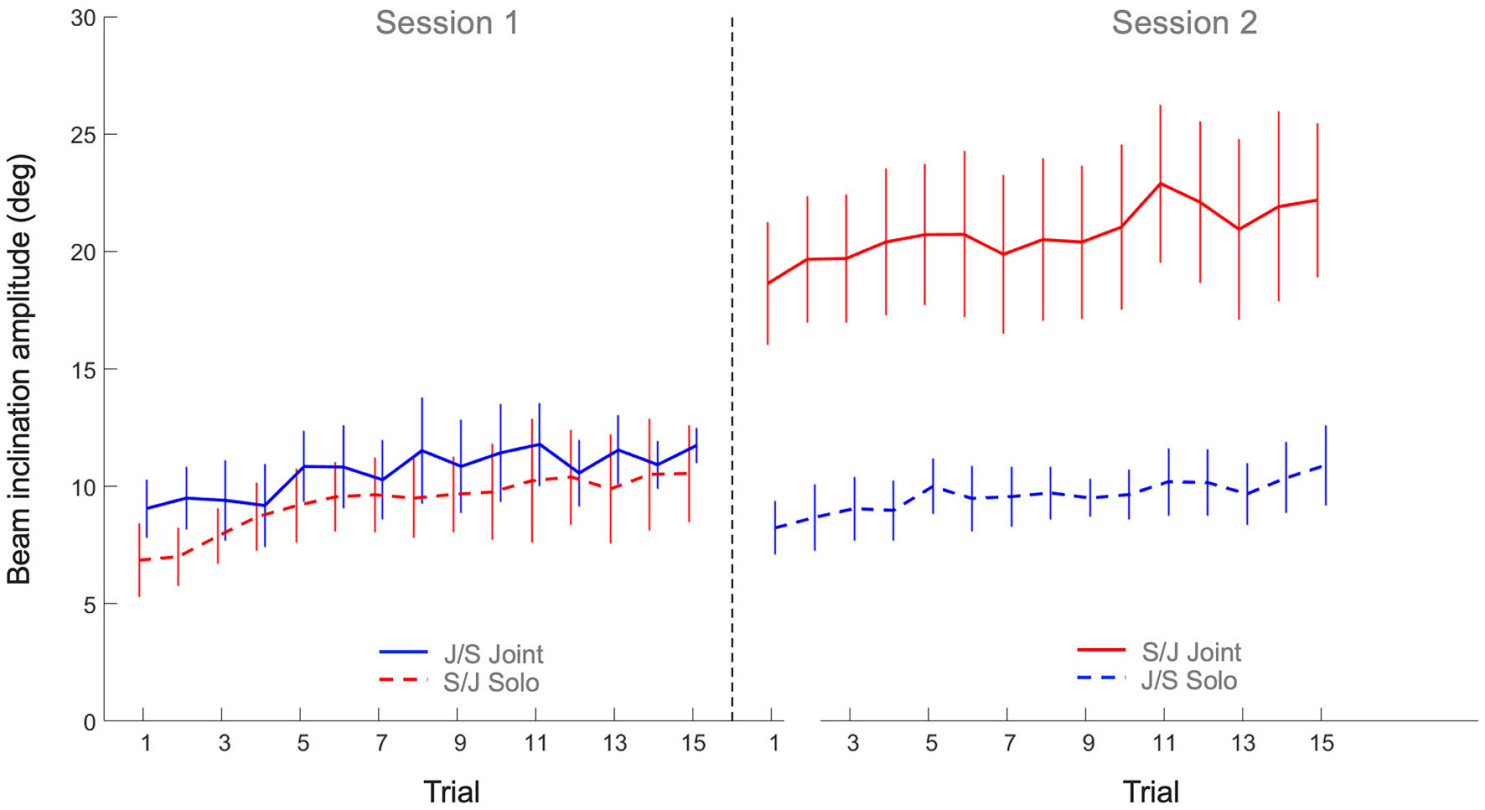
Mean beam angle amplitude (degrees) over practice trials per group and per task. Solid lines indicate joint action task performance, while dashed lines represent solo action task performances. Group S/J (red) first performed the solo action task (Session 1) and then the joint action task (Session 2), whereas Group J/S (blue) first performed the joint action task (Session 1) and then the solo action task (Session 2). Error bars represent between-participant standard deviations.

The 2 x 2 x 15 mixed design ANOVA on average beam inclination amplitude revealed significant main effects of Practice (*F*(3.87, 123.91) = 9.54, *p* < 0.001, η^2^_*p*_ = 0.23), Task (*F*(1, 32) = 24.3, *p* < 0.001, η^2^_*p*_ = 0.43) and Group (*F*(1, 32) = 15.0, *p* < 0.001, η^2^_*p*_ = 0.32), as well as a significant Task x Group interaction (*F*(1, 32) = 17.0, *p* < 0.001, η^2^_*p*_ = 0.35). All other effects were not significant. As can be seen in Figure 5, the main effect of Practice indicated that both groups increased the beam amplitude over trials on both tasks. *Post hoc* analysis of the Task x Group interaction revealed that Group S/J had a significantly (*p* < 0.001) larger beam amplitude on the joint action task (20.8 ± 6.4 degrees) than Group J/S (10.6 ± 3.0 degrees). Group S/J's beam amplitude on the joint action task was moreover significantly larger than the solo action amplitudes of Group S/J (9.3 ± 3.1 degrees, *p* < 0.001) and Group J/S (9.6 ± 2.2 degrees, *p* < 0.001). No other differences were significant.

### 3.3 Interpersonal coordination

**Figure 6:**
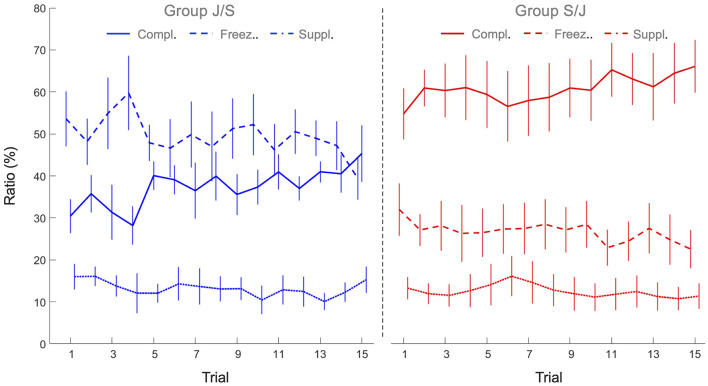
Mean freezing ratio (dashed lines), complementarity ratio (continuous lines) and supplementarity ratio (dotted lines) of the joint action task performances of group J/S (blue) and group S/J (red). Error bars represent between-participant standard deviations.

To capture the underlying interpersonal coordination, we analyzed the relative directions of vertical motion of both individuals, calculating how frequent (in proportion to total trial time) the two agents were moving in the same direction (supplementarity ratio), in the opposite direction (complementarity ratio) or, for at least one of the agents, not moving at all (freezing ratio). The 2 x 15 mixed design ANOVAs with Group (S/J, J/S) as between-participant factor and Practice (15 trials) as within-participant factor revealed significant main effects of Practice for all three ratios: As can be seen from Figure 6, the freezing ratio (*F*(14, 140) = 2.93, *p* < 0.001, $\eta_{p}^{2}$ = 0.23) and the supplementarity ratio (*F*(14, 140) = 1.83, *p* < 0.039, $\eta_{p}^{2}$ = 0.16) both decreased over practice, while the complementarity ratio increased over practice *F*(14, 140) = 3.10, *p* < 0.001, $\eta_{p}^{2}$ = 0.24). Moreover, the ANOVAs revealed significant main effects of Group for both the freezing ratio (*F*(1, 10) = 13.6, *p* < 0.004, $\eta_{p}^{2}$ = 0.58) and complementarity ratio (*F*(1, 10) = 15.5, *p* < 0.003, $\eta_{p}^{2}$ = 0.61). Freezing ratio was significantly lower in group S/J (26.7 ± 10.3 %) than in group J/S (49.6 ± 11.1 %), while the complementarity ratio, on the other hand, was higher in group S/J (60.7 ± 13.2%) than in group J/S (37.3 ± 6.4%). Supplementarity ratio was not significantly different between groups (S/J 12.5 ± 7.0%, J/S 13.2 ± 5.3%, *p* > 0.05). All other effects were not significant.

#### 3.3.1 Individual freezing

The freezing ratio as described above specifies how often at least one of the *two* individuals in a dyad froze their motion. But it does not indicate to what extent the freezing behavior of both agents overlap during the joint action. To assess this, we analyzed the individual freezing ratio's of both agents (i.e., the relative amount of time that an agent's handle had a velocity below the threshold of 0.05 m/s) and compared it to the freezing ratio of the dyad as a whole. When part of the individual freezing is overlapping, the dyadic freezing ratio must be lower than the sum of the individual ratios within a dyad. As shown in Table 1, the freezing ratio of all dyads was indeed lower than the sum of the individual ratios, implying some degree of overlap in freezing. The amount of overlap, however, was low in both groups (13.5% in group S/J and 14.3% in group J/S). Corroborated by visual inspection of the handle-motion time series, this finding indicates that the freezing of dyads was sequential rather than simultaneous. The sequentiality of the freezing behavior does not necessarily mean that the freezing was also equally distributed over the two agents. As a last step, we compared the two individual freezing ratios to assess the freezing distribution within dyads. As can be seen in the last column of Table 1, the difference between the two individual freezing ratios was again generally low. In group S/J, the difference was below 6% in all but one dyad (16.1%), while in group J/S –that demonstrated considerably higher dyadic freezing ratios– the differences were all below 22%. This suggests that freezing was more or less equally distributed within dyads, confirming the observed sequentially alternating character of the freezing behavior.

**Table 1:** Average freezing ratio (in percentage of total trial time) per dyad (D) and per agent (A) within a dyad during joint action task performance for group S/J and group J/S.

**Group**	**Dyad**	**D (%)**	**A1 (%)**	**A2 (%)**	**A1 + A2 (%)**	**D/(A1 + A2)**	**|A1 – A2| (%)**
S/J	1	38.5	18.0	23.0	41.0	0.94	5.0
	2	13.1	9.9	5.5	15.4	0.85	4.4
	3	17.3	9.3	11.4	20.7	0.84	2.1
	4	36.2	20.3	25.6	45.9	0.79	5.3
	5	31.4	26.0	9.9	35.9	0.88	16.1
	6	23.9	12.5	14.5	27.0	0.89	2.0
J/S	7	42.7	31.6	13.5	45.1	0.95	18.1
	8	64.7	48.7	33.7	82.4	0.79	15.0
	9	33.8	15.0	24.6	39.6	0.85	9.6
	10	53.0	26.7	32.6	59.3	0.89	5.9
	11	45.7	36.7	15.4	52.1	0.88	21.3
	12	57.5	29.9	43.9	73.8	0.78	20.0

## 4 Discussion

The goal of this study was to characterize the process of multi-agent motor learning. How do two individuals learn to coordinate their actions to acquire control in a novel joint action task? To investigate this, we designed the continuous manual ball-and-beam task in which agents had to control a ball rolling on a beam by manually adjusting the inclination of that beam. We asked participants to perform the task alone, in a solo action task setting, as well as in dyads, in a joint action task setting. For present purposes, we did not seek to relate dyads' performance on the joint action task to the corresponding individuals' performance on the solo action task, but focused on the development of performances over practice in both task settings. We found that participants significantly improved their performance over practice in both task settings, as evidenced by gradual increases in the scores over trials. Underlying this improvement in performance was a strong increase in ball speed and a moderate decrease in effective target width. The higher ball speeds were largely caused by increasing beam amplitudes over practice. We note that the observed increases in ball speed together with observed decreases in effective target width, indicating increases in accuracy, suggested an improvement in control over the ball and thereby provided a first indication of successful motor learning in both the solo and joint action task settings. From this perspective, the particularly high ball speeds attained by the S/J group on the joint action task are worth noticing. But how did participants, in particular the dyads in the joint action task setting, establish this improvement in performance over practice?

Based on Bernstein's (1967) theory of individual motor learning, we hypothesized that the first stage of multi-agent motor learning would be characterized by the freezing of systemic, task-relevant DFs to simplify control of the ball-and-beam system. Motion analyses of collaborating dyads in the joint action task confirmed this first hypothesis. In the early trials of dyads without any prior task experience (i.e., group J/S), more than half of the trial time (53% on average over first five trials; see Figure 6) consisted of the freezing of motion of at least one of the two individuals within a dyad. More specifically, dyads in the J/S group generally displayed a sequential, turn-taking pattern of interpersonal coordination to control the ball. Although these dyads increased the amount of beam movement over practice, the freezing ratio of all dyads in the J/S group remained relatively high throughout the joint action session (46% on average over last 5 trials). On a systemic level, the sequential motion of the two individuals implied that one of the two DFs of the ball-and-beam system was temporarily frozen during each half cycle of the ball. This suggests that the first stage of multi-agent motor learning may be considered *functionally equivalent* to the first phase of individual motor learning, as described by Bernstein (and corroborated empirically, e.g., Vereijken et al., 1992; Anderson and Sidaway, 1994; Hodges et al., 2005). By freezing task-relevant degrees of freedom, a system is constrained so as to be more controllable, regardless of whether this is a single- or a multi-agent system. As witnessed by the increase in performance score over practice, group J/S indeed progressed in terms of control in the joint action task.

Secondly, we hypothesized that freeing-up of systemic DFs could be stimulated by providing individuals with prior task experience in a constrained solo action task setting. To this end, we asked participants from the S/J group to practice a solo action task before practicing the joint action task and compared their performance to the J/S group that did not have any solo action experience before practicing the joint action task. As expected, we found that the (S/J) group with solo action task experience displayed a considerably lower amount of freezing of DFs (27% on average) during joint action than the (J/S) group without such experience (50% on average). Moreover, motion analyses demonstrated that S/J dyads moved the beam handles simultaneously and in opposite (i.e., complementary) directions for more than 60% of the time, compared to 37% for the J/S dyads. Such complementarity between agents in beam handle motions highlights the collaborative interpersonal coordination developed by dyads of the S/J group, with both individuals within a dyad (i.e., both available systemic DFs) cumulatively amplifying beam inclination adaptations. On a systemic level, compared to the J/S group, the much larger degree of complementarity between the motions of the individuals within the dyads allowed the S/J group to produce larger beam inclination amplitudes, associated with higher ball speeds that allowed to improve the scores. Over joint action practice, the S/J group's complementarity ratio increased (from 59% on the first five trials to 64% on the last five trials), while the freezing ratio simultaneously decreased (from 28% on the first five trials to 24% on the last five trials). While the J/S group showed a similar pattern of change (increase in complementarity ratio from 33% on the first five trials to 41% on the last five trials; decrease in freezing ratio from 53% on the first five trials to 46% on the last five trials), the dyadic coordination patterns remained qualitatively different between the two groups. Taken together, these results indicate that prior training on the constrained solo action task leads to subsequent freeing of systemic DFs during joint action, enabling the multi-agent system to create larger beam inclination amplitudes, exploit the passive forces in the system and improve the performance.

A question that comes up naturally when interpreting these results is whether a continuation of the joint action practice by the (J/S) group without any prior task experience would eventually lead to the same type of interpersonal coordination as demonstrated by the (S/J) group with prior solo action experience. On the one hand, the above-described increase in the complementarity ratio and decrease in the freezing ratio over joint action practice suggests that the interpersonal coordination would have continued to evolve with prolonged practice on the joint action task. This suggests that the performance scores that result from this coordination would also have continued to increase over practice. On the other hand, we recall that motor learning is not necessarily a gradual process. On the contrary, learning often progresses in a discontinuous, stage-like fashion (e.g., Vereijken et al., 1997). It is therefore not unlikely that some of the dyads would have continued to move sequentially for a long time before, if ever, making the transition to a more efficient form of complementary interpersonal coordination. In fact, this is exactly what Nalepka et al. (2017) found in their study of interpersonal coordination in a dyadic shepherding task. Over practice, some dyads soon switched from a suboptimal discrete strategy (“search and recover”) to a more efficient continuous solution (“coupled oscillatory containment”), while others continued the search and recover strategy throughout the entire duration of the experiment. Our results suggest a similar distinction between a discrete solution (i.e., sequential motion) and a continuous solution (simultaneous motion). Accordingly, we expect that following prolonged practice some dyads might have made the switch from sequential to simultaneous motion, while others might have continued in the suboptimal solution.

Finally, we evaluated whether joint action practice would lead to better performances in a subsequent solo action session of our ball-and-beam task. To test this, we asked the (J/S) group that started with a joint action task practice session to also engage in a solo action task practice session after that. We found that the solo action performance of this group was not significantly better than that of the (S/J) group that performed the solo action session without any prior task experience. We suggest that the key to understanding this finding might lie in the nature of what is learned during practice on this ball-and-beam task. To acquire control over the ball, one needs to learn how to relate the motion of the beam inclination to the motion of the rolling ball. In other words, the agent needs to learn how to handle a complex and non-linear relationship between the ball and the beam, mediated by gravitational and other passive forces in the system. In the solo action task setting, the agent-induced motion of the beam extremity is fully equivalent to changes in the beam inclination. This implies that an agent learns to couple motion of the beam extremity directly to the motion of the ball. In the joint action task setting, however, two agents independently change the beam inclination. For this reason, the agent not only needs to learn to couple the motion of its own beam extremity to the motion of the ball, but also to the other agent's motion. As discussed above, our results indicated that the multi-agent system approaches this highly challenging task by initially freezing one of its DFs in such a way that only one agent at a time changes the beam inclination. However, such freezing was not only not flawless (in the sense that it only implied handle motion of < 0.05 m/s), but was also only partial (60% of the time on average) and not necessarily always occurring at the exact same handle position. From the perspective of an individual, compared to the solo action task setting, such variations create additional noise that could be detrimental to learning the ball and beam relationship.

An alternative explanation for the absence of a transfer effect from joint action learning to solo action learning could be the sequential form of interpersonal coordination observed during the preceding joint action performances in this group. Agents generally froze their motion when the ball rolled in the direction of their partner and, in turn, started moving when the ball rolled back in their direction. By taking turns in controlling the ball instead of moving concurrently, individual agents only learned to control the ball during one half of the ball's full cycle. Yet, in the subsequent solo action setting agents were forced to control the ball throughout the full cycle instead of only during one half cycle. Speculatively, we suggest that the difference between sequential control in the joint action task and continuous control in the solo action task could be the principal reason for the apparent absence of a transfer effect in this ball-and-beam task. More generally, Karlinsky and Hodges (2018) also found that taking alternating turns in practicing a balancing task and observing someone else practicing that task did not improve the effectiveness of the motor learning process. This suggests that watching the other agent controlling the ball in between the agent's own actions was not beneficial to the motor learning process.

We conclude that motor learning of a challenging, novel joint action task is characterized by the initial freezing of task-relevant degrees of freedom so as to constrain the multi-agent system. Moreover, training in a constrained solo action setting stimulates freeing of DFs during a subsequent joint action performance, leading to significant improvements in joint action performance. Joint action training by itself, however, does not necessarily lead to better solo action performances in the same task. Overall, our results suggest that the distinguishable stages of multi-agent motor learning are functionally equivalent to those of individual motor learning, providing support for a systems approach to interpersonal coordination.

As a final point, we note that the ball-and-beam system has been extensively studied by control engineers as a simple laboratory model for nonlinear systems, focusing on the inherent instability of the ball's position on the beam. Feedback-based control schemes using a motor to drive changes in beam inclination angle are therefore typically developed for stabilizing the ball at a designated position (or series of positions) on the beam (e.g., Hauser et al., 1992; Rahmat et al., 2010; Bolívar-Vincenty and Beauchamp-Báez, 2014). Similar work has addressed its 2D extension to the ball-on-plate system (e.g., Fan et al., 2004; Debono and Bugeja, 2015). In the domain of human motor control, however, the manual control of such systems has so far received little to no attention (but see Huang et al., 2006; for an exception). We suggest that the continuous manual ball rolling task paradigm prefatorily explored in the present contribution can offer a rich window in the processes underlying perceptuomotor control and learning, in both solo and joint action settings. Indeed, the availability of system models for exploring the lawful relationship between ball and beam motion in principle allows addressing the task-level (i.e., ball motion) driven adaptations in beam motion characteristics in both single-agent and multi-agent settings.

## Data availability statement

The raw data supporting the conclusions of this article will be made available by the authors, without undue reservation.

## Ethics statement

The studies involving humans were approved by Comité d'Éthique pour la Recherche en STAPS (CERSTAPS), the French National Ethics Committee for Research in Sports and Movement Sciences. The studies were conducted in accordance with the local legislation and institutional requirements. The participants provided their written informed consent to participate in this study.

## Author contributions

MH: Conceptualization, Data curation, Formal analysis, Investigation, Methodology, Software, Writing – original draft, Writing – review & editing. RC: Conceptualization, Data curation, Formal analysis, Methodology, Software, Writing – review & editing. RB: Conceptualization, Funding acquisition, Investigation, Methodology, Project administration, Supervision, Validation, Writing – original draft, Writing – review & editing.
